# Prevalence of *BRCA1* and *BRCA2* gene mutations in Chinese patients with high‐risk breast cancer

**DOI:** 10.1002/mgg3.677

**Published:** 2019-04-09

**Authors:** Xiaozhen Wang, Haimeng Liu, Amina Maimaitiaili, Gang Zhao, Sijie Li, Zheng Lv, Di Wu, Aiping Shi, Xin Guan, Hongyao Jia, Menghan Li, Dong Song, Lihua Kang, Bing Han, Tong Fu, Ming Yang, Zhu Zhu, Ye Du, Yanqiu Song, Jinghui Hong, Zhimin Fan

**Affiliations:** ^1^ Department of Breast Surgery The First Hospital of Jilin University Changchun China; ^2^ Cancer Center of the First Hospital of Jilin University The First Hospital of Jilin University Changchun China

**Keywords:** *BRCA1*; *BRCA2*; mutation; breast cancer

## Abstract

**Background:**

Breast cancer is the most common cancer among women worldwide. Here, we report the prevalence of *BRCA1/2* mutations in patients with high‐risk breast cancer from Inner Mongolia and Jilin, China, which was a part of a nationwide project on the detection of *BRCA1/2* mutations in Chinese patients with hereditary breast cancer.

**Methods:**

According to the criteria, index patients from a total of 245 independent families were initially recruited. All 49 exons of *BRCA1* and *BRCA2* and adjacent noncoding regions were screened for mutations based on next‐generation sequencing from collected saliva.

**Results:**

We detected 17 *BRCA1/2* variants in 18 of 216 (8.3%) index patients with high‐risk breast cancer. Among these, seven mutations were novel, including four *BRCA1* mutations (c.123_124delCAinsAT, c.5093_5096delCTAA, c.5396‐2A>G, and c.2054delinsGAAGAGTAACAAGTAAGAAGAGTAACAAGAAG), and three *BRCA2* mutations (c.304A>T, c.7552_7553insT, and c.9548_9549insA). The *BRCA1/2* variants were identified in 14% (8/57) of the patients with triple‐negative breast cancer and in 6.3% (10/159) of the patients with non‐triple‐negative breast cancer. There was no significant difference between the two groups (*p* = 0.07). A higher frequency for *BRCA1* mutations was observed in patients with triple‐negative breast cancer than in those with non‐triple‐negative breast cancer (12.3% vs. 2.5%, *p* = 0.004). The frequencies of the *BRCA2* mutations were not significantly different between patients with triple‐negative breast cancer and those with non‐triple‐negative breast cancer (1.8% vs. 3.8%, *p* = 0.46).

**Conclusion:**

We found that patients with triple‐negative breast cancer had a higher frequency of *BRCA1* mutations than those with non‐triple‐negative breast cancer. In this study, no significant associations between the *BRCA1/2* mutation status and age, family history of breast cancer, ovarian cancer, pancreatic cancer and prostate cancer, number of primary lesions, tumor size, or lymph node metastasis were observed.

## 1 BACKGROUND

Breast cancer is the most common cancer among women worldwide, including China (Fan et al., 2014; Ferlay et al., 2015). In recent years, the incidence of breast cancer in China has increased, accounting for 12.2% of all newly diagnosed cases and 9.6% of all deaths from breast cancer worldwide (Fan et al., 2014). The *BRCA1* (OMIM accession number 113705) and *BRCA2* (OMIM accession number 600185) (*BRCA1/2*) genes are the earliest discovered and most studied genes that are related to breast cancer. *BRCA1/2* mutations are identified in approximately 10% of breast cancers (Nik‐Zainal et al., 2016) and 8%–13% of all ovarian cancer cases (Liu et al., 2012). Compared to the general population, the risk of breast cancer in *BRCA1* and *BRCA2* mutation carriers increases by 10‐ to 20‐fold (Ludwig, Neuner, Butler, Geurts, & Kong, 2016; Nelson, Huffman, Fu, Harris, & U.S. Preventive Services Task Force, 2005). Compared to the general population, the risk of recurrence of contralateral breast cancer in patients with *BRCA1/2* mutation increases by two to sixfold, and the younger the age at onset, the greater the risk of recurrence (Domchek & Kaunitz, 2016).

Although there are several studies on *BRCA1/2* mutations in Chinese patients with hereditary breast cancer (Kim et al., 2016; Li et al., 2019; Liang et al., 2018; Liu et al., 2017; Wang et al., 2017, 2018; Wei et al., 2018; Xie, Gou, Wang, Zhong, & Zheng, 2017), the sample sizes in many of these studies are relatively small (Liu et al., 2017; Wang et al., 2017; Xie et al., 2017). In addition, no study has been conducted in patients with breast cancer from the Northern Frontier and Northeast regions of China to date. In this study, we report the prevalence of *BRCA1/2* mutations in patients with high‐risk breast cancer from Inner Mongolia and Jilin, China, as part of a nationwide project on the detection of *BRCA1/2* mutations in Chinese patients with hereditary breast cancer, led by the 307th Hospital of the Chinese People's Liberation Army and involving a number of breast cancer clinics across the country. The overall goal of this project included (a) screening of a large sample of Chinese high‐risk breast cancer populations for *BRCA1/2* mutations; (b) establishing a database of *BRCA1/2* mutations in Chinese breast cancer populations; and (c) establishing a risk model of breast cancer that is associated with *BRCA1/2* mutations in Chinese populations.

## 2 METHODS

### 2.1 Patients

Our research has been approved by the ethics committee, under the “Ethical Compliance”, we started the project. According to the U.S. National Comprehensive Cancer Network Genetic/Family High Risk Assessment: Breast and Ovarian Cancer Clinical Practice Guidelines in Oncology (Gradishar et al., 2018), we screened for breast cancer families with high‐risk breast cancer in Inner Mongolia and Jilin, China. All patients were diagnosed with breast cancer after 2010, except that diagnosis time was unlimited in the case of typical familiar patients. One index patient was selected from each independent family, with preference for the probands. After the *BRCA1/2* mutations were identified in the index patients, their first‐ and second‐degree relatives were screened.

The index patients met one or more of the following inclusion criteria: (a) age at diagnosis ≤45 years; (b) age at diagnosis ≤50 years and the presence of ≥2 primary lesions; and (c) meeting one or more of the following family histories: i) age at diagnosis ≤50 years and ≥1 close relative with breast cancer; ii) diagnosed at any age and ≥1 close relative with breast cancer whose age at diagnosis ≤50 years; iii) diagnosed at any age and ≥2 close relatives with breast cancer; iv) diagnosed at any age and ≥1 close relative with epithelial ovarian cancer; v) diagnosed at any age and ≥2 close relatives with pancreatic cancer, and/or prostate cancer (Gleason score greater than 7, at any age); vi) diagnosed at any age, and ≥1 male close relative with breast cancer; (d) triple‐negative breast cancer and the age at onset was not more than 60 years; and (e) male breast cancer.

First‐ and second‐degree relatives of the *BRCA1/2* mutation carriers were enrolled only when the mutations in the index patients were confirmed by Sanger sequencing. One to five relatives from each family were enrolled as follows: (a) first‐ and second‐degree female adult relatives (≥18 years) were selected from the same side of the paternal or maternal line according to the family's incidence and (b) first‐ and second‐degree male breast cancer relatives.

All inpatients and review outpatients in the Department of Breast Surgery assessed by our hospital from April 2010 to March 2017 were recruited into this study. All patients underwent surgical treatment. According to the above inclusion criteria, index patients from a total of 245 independent families were initially recruited. An additional eight first‐ and second‐degree relatives of three index patients who carried the *BRCA1/2* mutations were further enrolled, including the father and mother of Patient 033A; the father, mother, older sister, younger sister, and daughter of Patient 073A; and the mother of Patient 196A. There was no restriction on race and ethnic group during patient enrollment. Approximately 240 patients underwent surgery in our hospital, and five patients were review outpatients in our hospital but underwent surgery in other hospitals.

Among the 245 independent families, 12 patients with clinical samples of poor quality and 17 patients who did not meet the criteria for index patients were excluded from this study. A total of 216 index patients were finally enrolled in this study.

This study was registered at ClinicalTrials.gov (NCT02593435; The Registration Program of *BRCA1/2* Gene in Chinese Breast Cancer Group). Written informed consent for *BRCA1/2* gene testing was obtained from all patients, and the study was approved by the Ethics Committee of the First Hospital of Jilin University (Approval No. ky‐2015‐10‐32).

### 2.2 DNA extraction and mutation analysis

Approximately, 2‐ml of saliva was collected in an anticoagulant tube containing the nucleic acid preservation solution and stored at 4°C until use. Genomic DNA was extracted using the TIANamp Genomic DNA Extraction Kit (Tiangen Biochemical Technology Co., Ltd., Beijing, China).

Genomic DNA was fragmented using a Bioruptor sonication device (Diagenode, Leige, Belgium). A DNA fragment library was constructed according to the Illumina standard protocol (Illumina, San Diego, CA). All exons of breast cancer‐related genes were captured with the NimbleGen SeqCap EZ Choice library according to the Roche standard protocol (Roche, Shanghai, China). The quantity of the library was determined with a Bioanalyzer (Agilent, Santa Clara, CA). Paired‐end 100‐bp next‐generation sequencing (NGS) was performed on an Illumina HiSeq 2500 platform (Illumina). A total of 16,000 bp spanning all 49 exons of *BRCA1* and *BRCA2* and adjacent noncoding regions were sequenced. The depth of coverage was over 300×.

Sequence reads were aligned to the human reference genome (hg19) using the Burrows–Wheeler Aligner software (BWA, version 0.5.9) (Li & Durbin, 2009). Duplicate reads were marked with Picard (http://broadinstitute.github.io/picard/). Realignment and recalibration of the reads were performed with the Genome Analysis Toolkit (GATK; version 3.0) (McKenna et al., 2010). Single‐nucleotide polymorphism and insertion/deletion variants were called with the HaplotypeCaller in GATK 3.0 (McKenna et al., 2010). The variants were annotated by a modified ANNOVAR pipeline (Wang, Li, & Hakonarson, 2010).

The index patients were initially screened for *BRCA1/2* mutations using NGS, and the identified mutations were verified using Sanger sequencing. The relatives of index patients were further screened for the *BRCA1/2* mutations identified in the index patients using Sanger sequencing.

The GenBank reference sequence and version number for *BRCA1* is NM_007294.3, and for *BRCA2* is NM_000059.3. Gene mutations were named according to the Human Genome Variation Society nomenclature. The interpretation of *BRCA1/2* variations was based on the Sequence Variation Interpretation Standards and Guidelines (2015 Edition) of the American College of Medical Genetics and Genomics (ACMG) and the Association for Molecular Pathology. The detected mutations were compared to the Breast Cancer Information Core (BIC) database (http://research.nhgri.nih.gov/Bic/) and the ClinVar database (http://www.ncbi.nlm.nih.gov/clinvar/) to determine whether it is a novel mutation.

According to the International Agency for Research Cancer (Plon et al., 2008), ACMG (Kleinberger, Maloney, Pollin, & Jeng, 2016; Li et al., 2017; Richards et al., 2008, 2015), and the Evidence‐based Network for the Interpretation of Germline Mutation Alleles (ENIGMA, http://enigmaconsortium.org/) (Colombo et al., 2014; Thomassen et al., 2012; Thompson et al., 2014), the detected variants in the *BRCA1/2* gene were classified into the following five categories: pathogenic (category 5, likelihood of disease >0.99), possibly pathogenic (category 4, likelihood of disease between 0.95 and 0.99), unknown pathogenicity (category 3, likelihood of disease between 0.05 and 0.949), possibly benign (category 2, likelihood of disease between 0.01 and 0.049), and benign (category 1, likelihood of disease <0.01).

### 2.3 Statistical analysis

Statistical analysis was performed using Statistical Product and Service Solutions 24.0 statistical software (IBM Corp., Armonk, NY). The distribution of *BRCA1/2* variants in patients with triple‐negative and non‐triple‐negative breast cancer were compared using the χ^2^ test. The associations of the *BRCA1/2* mutations with clinical features of the patients were evaluated using the χ^2^ test or Fisher's exact test. Differences with *P* values of <0.05 were considered statistically significant.

## 3 RESULTS

### 3.1 Demographic and clinical characteristics of the patients

Among the 216 index patients, 47 patients had a family history of breast cancer, epithelial ovarian cancer, pancreatic cancer, and/or prostate cancer. The median age at diagnosis was 42 years (range: 21–67 years). Approximately 175 patients had an age at diagnosis ≤45 years (43 patients ≤35 years, 52 patients >35 years and ≤40 years, and 80 patients >40 years and ≤45 years). Seven patients had more than two primary tumors, three of whom had bilateral breast cancer. Two breast cancer patients were male. Based on immunohistochemistry, 216 patients were divided into three groups, including 57 patients with triple‐negative breast cancer and 159 patients with non‐triple‐negative breast cancer. There were 200 and 16 patients from Jilin and Inner Mongolia, respectively, including 202 Han patients, nine Mongolian patients, two Korean patients, two Manchu patients, and one Hui patient.

### 3.2 Prevalence of *BRCA1/2* variants in patients with high‐risk breast cancer

A total of 17 *BRCA1/2* mutations were detected in 18 of 216 (8.3%) index patients with high‐risk breast cancer. All patients carrying the *BRCA1/2* mutations were Han Chinese. Among these 17 mutations, eight mutations were novel and have not been reported in the BIC and/or ClinVar databases, including five *BRCA1* mutations (Table 1) and three *BRCA2* mutations (Table 2).

**Table 1:** Distribution of *BRCA1* * mutations in 216 patients with high‐risk breast cancer

Patient ID	Nucleotide change	Exon	Amino acid change	Mutation type	Pathogenicity	BIC	ClinVar	Age at diagnosis (years)	Cancer family history	Triple‐negative breast cancer
091A	c.123_124delCAinsAT	1	p.His41_Ile42delinsGlnLeu	Missense	Likely pathogenic	No	No	44	Mother lung cancer	Yes
042A	c.1934delC	11	p.Ser645Leufs*6	Missense	Pathogenic	No	No	39	No	Yes
219A	c.2054delinsGAAGAGTAACAAGTAAGAAGAGTAACAAGAAG	11	p.Asn685Argfs*12	Missense	Pathogenic	No	No	38	No	No
036A	c.2138C>G	11	p.Ser713Ter	Nonsense	Pathogenic	Yes	Yes	44	No	No
082A	c.2572C>T	11	p.Gln858Ter	Nonsense	Pathogenic	No	Yes	45	Mother breast cancer and colorectal cancer	Yes
079A	c.2751delC	11	p.Lys918Serfs*82	Missense	Pathogenic	No	Yes	31	Aunt endometrial cancer	Yes
198A	c.3841C>T	11	p.Gln1281Ter	Nonsense	Pathogenic	Yes	Yes	39	No	Yes
139A	c.3916_3917delTT	12	p.Leu1306Aspfs*23	Missense	Pathogenic	Yes	Yes	29	Aunt ovarian cancer; uncle stomach cancer; cousin thyroid cancer	No
196A	c.5093_5096delCTAA	—	p.Thr1698Ilefs*2	Missense	Pathogenic	No	No	33	Mother and aunts breast cancer; grandfather stomach cancer	No
054A	c.5194−2A>G	—	—	Splice site	Pathogenic/Likely pathogenic	Yes	Yes	42	Mother ovarian cancer	Yes
202A	c.5396−2A>G	—	—	Splice site	Likely pathogenic	No	No	37	No	Yes

BIC: breast cancer information core.

* *BRCA1* (NM_007294.3).

**Table 2:** Distribution of *BRCA2* * mutations in 216 patients with high‐risk breast cancer

Patient ID	Nucleotide change	Exon	Amino acid change	Mutation type	Pathogenicity	BIC	ClinVar	Age at diagnosis (years)	Cancer family history	Triple‐negative breast cancer
033A	c.304A>T	3	p.Lys102Ter	Nonsense	Pathogenic	No	No	28	Father pancreatic cancer	No
192A	c.464_468delGAGAT	5	p.Arg155Lysfs*26	Missense	Unknown	No	Yes	45	No	No
073A	c.5959C>T	11	p.Gln1987Ter	Nonsense	Pathogenic	Yes	Yes	47	Father pancreatic cancer; older sister breast cancer	No
212A	c.5959C>T	11	p.Gln1987Ter	Nonsense	Pathogenic	Yes	Yes	36	No	No
064A	c.7552_7553insT	15	p.Pro2519Alafs*20	Missense	Pathogenic	No	No	33	No	No
140A	c.8364G>A	—	p.Trp2788Ter	Nonsense	Pathogenic	No	Yes	42	No	Yes
141A	c.9548_9549insA	—	p.Leu3184Thrfs*5	Missense	Pathogenic	No	No	41	Father bladder cancer	No

BIC: breast cancer information core.

* *BRCA2* (NM_000059.3).

Eleven *BRCA1* pathogenic mutations were detected in 11 (5.1%) of the 216 patients. Six mutations were known mutations that have been reported in the BIC and/or ClinVar databases, including c.2138C>G, c.2751delC, c.2572C>T, c.3916_3917delTT, c.3841C>T, and c.5194‐2A>G. The other five mutations have not been reported in the BIC and/or ClinVar databases, which included c.1934delC, c.123_124delCAinsAT, c.5093_5096delCTAA, c.5396‐2A>G, and c.2054delinsGAAGAGTAACAAGTAAGAAGAGTAACAAGAAG (Table 1).

Six *BRCA2* pathogenic variants were detected in seven (3.2%) of the 216 patients. Three variants were previously reported, including c.5959C>T, c.8364G>A, and c.464_468delGAGAT. Two patients carried the c.5959C>T mutation. The other three variants were novel, including c.304A>T, c.7552_7553insT, and c.9548_9549insA (Table 2).

### 3.3 Distribution of *BRCA1/2* variants in patients with triple‐negative and non‐triple‐negative breast cancer

The *BRCA1/2* variants were identified in 14% (8/57) of the patients with triple‐negative breast cancer and in 6.3% (10/159) of the patients with non‐triple‐negative breast cancer (Table 3). There was no significant difference between the two groups (*p* = 0.07). A higher frequency for *BRCA1* mutations was observed in patients with triple‐negative breast cancer than in those with non‐triple‐negative breast cancer (12.3% vs. 2.5%, *p* = 0.004). The frequencies of the *BRCA2* mutations were not significantly different between patients with triple‐negative breast cancer and those with non‐triple‐negative breast cancer (1.8% vs. 3.8%, *p* = 0.46).

**Table 3:** Distribution of *BRCA1/2* * mutations in patients with triple‐negative and non‐triple‐negative breast cancer

Gene	Number of triple‐negative breast cancer patients harboring variants (*n* = 57)	Number of non‐triple‐negative breast cancer patients harboring variants (*n* = 159)	*p*
*BRCA1*	7 (12.3%)	4 (2.5%)	0.004
*BRCA2*	1 (1.8%)	6 (3.8%)	0.46
*BRCA1/2*	8 (14.0%)	10 (6.3%)	0.07

* *BRCA1* (NM_007294.3) and* BRCA2* (NM_000059.3).

### 3.4 *BRCA1/2* phenotype‐genotype correlations

No significant association was found between the *BRCA1/2* mutation status and age; family history of breast cancer, ovarian cancer, pancreatic cancer and prostate cancer; number of primary lesions; tumor size; and lymph node metastasis (all *p* > 0.05; Table 4). The distribution of the identified *BRCA1/2* variants in family members of the index patients is shown in Table 5.

**Table 4:** *BRCA1/2* * genotype‐phenotype correlations

Feature	Number of patients	Patients with variants, *n* (%)	Patients without variants, *n* (%)	*p*
N	216	18 (8.3)	198 (91.7)	
Age at diagnosis (year)				0.28
≤35	43	6 (14.0)	37 (86.0)	
35–40	52	5 (9.6)	47 (90.4)	
40–45	80	6 (7.5)	74 (92.5)	
>45	41	1 (2.4)	40 (97.6)	
Cancer family history				0.52
Yes	47	5 (10.6)	42 (89.4)	
No	169	13 (7.7)	156 (92.3)	
≥2 primary lesions				0.61
Yes	7	1 (14.3)	6 (85.7)	
No	209	17 (8.1)	192 (91.9)	
Tumor size (cm)				0.42
In situ cancer	6	0 (0.0)	6 (100.0)	
≤2	133	12 (9.0)	121 (91.0)	
>2	70	4 (5.7)	66 (94.3)	
Lymph node metastasis				0.59
Yes	87	9 (10.3)	78 (89.7)	
No	121	8 (6.6)	113 (93.4)	

* *BRCA1* (NM_007294.3) and* BRCA2* (NM_000059.3).

**Table 5:** Distribution of *BRCA1/2* * variants in family members of the index patients

Gene	Index patient ID	Family member ID	Nucleotide change	Amino acid change	Relationship to the index patient	Cancer history of the family member	Age at diagnosis (y)
*BRCA1*	196A	196B	c.5093_5096delCTAA	p.Thr1698Ilefs*2	Mother	Breast cancer	61
*BRCA2*	033A	033B	No	—	Mother	No	—
		033C	c.304A>T	p.Lys102Ter	Father	Pancreatic cancer	50
*BRCA2*	073A	073B	No	—	Father	Pancreatic cancer	50
		073C	No	—	Older sister	Breast cancer	45
		073D	No	—	Mother	No	—
		073E	No	—	Younger sister	No	—
		073F	No		Daughter	No	—

* *BRCA1* (NM_007294.3) and* BRCA2* (NM_000059.3).

## 4 DISCUSSION

BRCA1/2 protein plays an important role in nonhomologous end joining (NHEJ) repair, homologous recombination repair, cell cycle regulation, gene transcription regulation, and chromatin stability after DNA damage (Clark, Rodriguez, Snyder, Hankins, & Boehning, 2012). The N‐terminus of the BRCA1 protein contains a really interesting new gene domain and a nuclear localization sequence (NLS), and the C‐terminus contains two coiled coil domains (nucleic acid sequences c.3759‐3819 and c.4191‐4272), one SQ/TQ cluster domain (SCD, amino acid residues 1,280–1,524) and two BRCA1 C‐terminal (BRCT) domains (nucleic acid sequences c.4926‐5169 and c.5268‐5526) (Clark et al., 2012). The coiled coil domain is a protein‐binding region of BRCA1 protein and BRCA2‐associated protein (partner and localizer of BRCA2, PALB2) (OMIM accession number 610355), which form the BRCA1‐PALB2‐BRCA2 complex. The SCD region contains approximately 10 ataxia‐telangiectasia mutated (ATM) (OMIM accession number 607585) phosphorylation loci. The BRCT domain can bind to ATM‐phosphorylated abraxas, CTBP‐interacting protein (RBBP8; OMIM accession number 604124), and BRCA1 interacting protein C‐terminal helicase 1 (OMIM accession number 605882) and participate in homologous recombination repair after DNA damage (Christou & Kyriacou, 2013; Roy, Chun, & Powell, 2011).

In this study, 11 *BRCA1* mutations were detected in Chinese patients with breast cancer. Four of these mutations (c.1934delC, c.2138C>G, c.2572C>T, and c.3916_3917delTT) have been reported in Chinese populations (Li et al., 2019; Liang et al., 2018; Wei et al., 2018). Five *BRCA1* variants have not been reported in the BIC and/or ClinVar databases. Among these mutations, c.5093_5096delCTAA and c.2054delinsGAAGAGTAACAAAAAAGAAGAGTAACAAGAAG were pathogenic mutations, whereas 123_124delCAinsAT and c.5396‐2A>G were predicted to be pathogenic. However the number of samples is too small, we cannot propose that the novel pathogenic mutations might be specific to the Chinese population. Also we are unable to determine the exact relationship between these new pathogenic mutations and breast cancer, so more samples analysis and long‐term follow‐up are still needed, and further molecular biology experiment for mutations will have important significance in the future, which will help us get more study results. The c.1934delC mutation changes serine to leucine at the 645th amino acid of the BRCA1 protein, resulting in premature protein truncation at the sixth amino acid of the new reading frame. The c.5093_5096delCTAA variant results in a substitution of threonine to isoleucine at the 1698th amino acid of the BRCA1 protein, leading to premature protein truncation at the second amino acid of the new reading frame. The c.2054delinsGAAGAGTAACAAGTAAGAAGAGTAACAAGAAG variant is predicted to result in the substitution of asparagine to arginine at the 685th amino acid of BRCA1 protein, which results in premature protein truncation at the 12th amino acid of the new reading frame. In silico prediction analysis suggests that these three variants may cause nonsense‐mediated mRNA decay (NMD), leading to loss of BRCA1 protein expression. Studies have shown that the early termination of *BRCA1* sequence translation can cause abnormal expression of BRCA1 protein, resulting in the development of breast cancer (Christou & Kyriacou, 2013; Clark et al., 2012; Roy et al., 2011). Therefore, these mutations are considered to be pathogenic. In addition, the 123_124delCAinsAT mutation causes a deletion of histidine at the 41th amino acid and isoleucine at 42th amino acid and an insertion of glutamine and leucine. His41 is a highly conserved amino acid in BRCA1, and another variant at this codon, p.His41Arg, is listed as a pathogenic variant in ClinVar. Therefore, the 123_124delCAinsAT variant is considered to be potentially pathogenic. The c.5396‐2A>G variant is predicted to disrupt normal splicing of the *BRCA1* gene, leading to abnormal protein production. This variant is thus classified as likely pathogenic.

The novel *BRCA1* variant c.5093_5096delCTAA identified in this study is located in the BRCT exon region. The index patient who carried this mutation was diagnosed with breast cancer at the age of 33. Her mother carried the same mutation and was diagnosed with breast cancer at the age of 61 (Table 5). In addition, her aunts also suffered from breast cancer, while her grandfather suffered from gastric cancer. Recently, Moghadasi and colleagues estimated that the cumulative risk of breast and ovarian cancer by the age of 70 years was 20% and 6%, respectively, in the carriers with *BRCA1* mutation c.5096G>A (p.Arg1699Gln) (Moghadasi et al., 2018). In addition, Buzolin et al. reported that the *BRCA1* mutation c.5095C>T (p.Arg1699Trp) was a pathogenic mutation (Buzolin et al., 2017). Collectively, these findings support that the c.5093_5096delCTAA variant is pathogenic and may be a founder mutation in the Chinese population.

Two *BRCA1* splice site mutations, c.5194‐2A>G and c.5396‐2A>G, identified in this study are located in introns 18 and 21 of the BRCT, respectively, which may affect the normal splicing of the *BRCA1* gene, resulting in an altered structure of the BRCA1 protein, making it unable to perform normal DNA repair functions, eventually leading to an increased risk for tumorigenesis. Three *BRCA1* mutations (c.2138C>G, c.1934delC, and c.2054delinsGAAGAGTAACAAGTAAGAAGAGTAACAAGAAG) are located in the region where BRCA1 binds to RAD50 (OMIM accession number 604040). After BRCA1 binds to RAD50, the Rad50/MreII/NbsI complex is recruited to the DNA double‐strand break site, making it easy to repair DNA damage, particularly NHEJ repair (Clark et al., 2012). The *BRCA1* c.2751delC and c.2572C>T variants are located in the region where BRCA1 interacts with RAD51 (OMIM accession number 179617). During cell mitosis and meiosis, BRCA1 binds to RAD51, and RAD51 binds to single‐stranded DNA (ssDNA), facilitating homologous recombination to repair HR (Clark et al., 2012). The *BRCA1* c.3916_3917delTT and c.3841C>T mutations are located in the SCD region, which can be phosphorylated by ATM/ATR, and then the phosphorylated BRCA1 is recruited to the double‐strand break site for DNA damage repair (Clark et al., 2012).

In this study, six *BRCA2* mutations were detected in Chinese patients with breast cancer. An important function of the BRCA2 protein is to mediate homologous recombination repair after DNA damage. The important functional structure of this protein includes the N‐terminal binding to the PALB2 protein (amino acid residues 21‐39), the BRC domain (containing eight BRC repeats, amino acid residues 1009‐2083), the DNA binding domain (DBD), and the C‐terminus comprising the NLS and cyclin‐dependent kinase (Roy et al., 2011). The DBD comprises a helical domain and three oligonucleotide binding domains, and its main function is to bind single‐stranded or double‐stranded DNA. The BRC domain and the C‐terminus can bind to the recombinant enzyme RAD51 and bind to single‐stranded or double‐stranded DNA through the DBD, thereby performing homologous recombination repair after DNA damage (Roy et al., 2011).

Two patients in this study harbored the c.5959C>T variant in the *BRCA2* gene, which has been reported in the BIC and/or ClinVar. This variant is located within the BRC domain, an important functional domain of BRCA2 protein and is predicted to result in the disruption of BRCA2 protein expression and the loss of homologous recombination repair. One of the patients with the c.5959C>T variant was diagnosed with breast cancer at the age of 47. Although his father was diagnosed with pancreatic cancer at the age of 50, and his older sister was diagnosed with breast cancer at the age of 45, this mutation was not detected in his father, older sister, mother, younger sister, or daughter (Table 5). Liang et al. recently reported on a Chinese patient who harbored the *BRCA2* c.5959C>T variant that was diagnosed with breast cancer at the age of 53 and had a family history of breast cancer (Liang et al., 2018).

Three *BRCA2* variants (c.304A>T, c.7552_7553insT, and c.9548_9549insA) detected in this study were novel (i.e. have not been reported in the literature and have not been recorded in the BIC and ClinVar databases). A frameshift variant c.304_304delA (p.Leu103fs) has been reported in Chinese patients (Liang et al., 2018). This indicates that this site may be a common mutation site in the Chinese population. The c.304A>T mutation is predicted to result in protein truncation involving the amino acid lysine at position 102 of the BRCA2 protein. The c.7552_7553insT is predicted to result in the substitution of the amino acid proline to alanine at position 2519 of *BRCA2* and introduces a stop codon at the 20th amino acid of the new reading frame. The *BRCA2*c.9548_9549insA mutation is predicted to result in the substitution of the amino acid leucine to threonine at position 3184th of the BRCA2 protein, introducing a stop codon at position 5 of the new reading frame. Prediction analysis indicates that these variants may cause NMD, leading to the loss of BRCA2 protein expression, and therefore, these variants are considered pathogenic. The *BRCA2* c.304A>T carrier in this study was diagnosed with breast cancer at the age of 28. His father harbored the same mutation and was diagnosed with pancreatic cancer at the age of 50. His mother did not carry this mutation and had no cancer (Table 5).

In this study, we found that patients with triple‐negative breast cancer had a higher *BRCA1* mutation rate than those with non‐triple‐negative breast cancer. Our results agree with those of previous studies in Chinese patients with breast cancer (Li et al., 2019; Liang et al., 2018; Wei et al., 2018). We did not find significant associations between *BRCA1/2* mutation status and age; family history of breast cancer, ovarian cancer, pancreatic cancer, and prostate cancer; number of primary lesions; tumor size; or lymph node metastasis.

BRCA1/2 plays an important role in the mechanism of homologous recombination repair after DNA damage, and *BRCA1/2* mutations may cause a significant increase in genomic instability. The *BRCA1/2* germline mutations can significantly increase the risk for breast, ovarian, and other cancers in women (Mafficini et al., 2016). Recent studies have found that *BRCA1/2* somatic mutations can also be detected in tumor tissues of breast or ovarian cancer (George, Banerjee, & Kaye, 2017; Moschetta, George, Kaye, & Banerjee, 2016). Patients with ovarian cancer who carry *BRCA1/2* mutations are very sensitive to platinum‐based chemotherapy with good prognosis (Byrski et al., 2012), and can benefit from treatment with poly ADP‐ribosome polymerase inhibitors (Isakoff, 2010; Rouleau, Patel, Hendzel, Kaufmann, & Poirier, 2010; Ström et al., 2011). Clinical studies have shown that prophylactic bilateral mammectomy or bilateral oophorectomy can reduce 50%–90% of the risk of breast and ovarian cancer in women who have family history of breast cancer and carry *BRCA1/2* mutations (Domchek & Kaunitz, 2016; Rizvi, Truong, & Truong, 2017). With the advancement of precision medicine and targeted therapy, detection of *BRCA1/2* mutations in blood and/or tumor tissues of patients with breast and ovarian cancer will help select targeted drugs and chemotherapy regimens and better assess prognosis (Robson et al., 2017).

In summary, we detected 17 *BRCA1/2* mutations in 18 of 216 (8.3%) index patients with high‐risk breast cancer. Among these, eight mutations were novel, including five *BRCA1* mutations and three *BRCA2* mutations. We found that patients with triple‐negative breast cancer had a higher *BRCA1* mutation rate than those with non‐triple‐negative breast cancer. We did not find any significant association between *BRCA1/2* mutation status and age; family history of breast cancer, ovarian cancer, pancreatic cancer, and prostate cancer; number of primary lesions; tumor size; and lymph node metastasis.

## CONFLICT OF INTEREST

The authors declare that they have no competing interests.
